# Targeted Amplicon Sequencing for Single-Nucleotide-Polymorphism Genotyping of Attaching and Effacing Escherichia coli O26:H11 Cattle Strains via a High-Throughput Library Preparation Technique

**DOI:** 10.1128/AEM.03182-15

**Published:** 2016-01-07

**Authors:** Sarah A. Ison, Sabine Delannoy, Marie Bugarel, Tiruvoor G. Nagaraja, David G. Renter, Henk C. den Bakker, Kendra K. Nightingale, Patrick Fach, Guy H. Loneragan

**Affiliations:** a Department of Animal and Food Sciences, Texas Tech University, Lubbock, Texas, USA; b ANSES Food Safety Laboratory, Platform IdentyPath, Université Paris-Est, Maisons-Alfort, France; c Department of Diagnostic Medicine Pathobiology, Kansas State University, Manhattan, Kansas, USA

## Abstract

Enterohemorrhagic Escherichia coli (EHEC) O26:H11, a serotype within Shiga toxin-producing E. coli (STEC) that causes severe human disease, has been considered to have evolved from attaching and effacing E. coli (AEEC) O26:H11 through the acquisition of a Shiga toxin-encoding gene. Targeted amplicon sequencing using next-generation sequencing technology of 48 phylogenetically informative single-nucleotide polymorphisms (SNPs) and three SNPs differentiating Shiga toxin-positive (*stx*-positive) strains from Shiga toxin-negative (*stx*-negative) strains were used to infer the phylogenetic relationships of 178 E. coli O26:H11 strains (6 *stx*-positive strains and 172 *stx*-negative AEEC strains) from cattle feces to 7 publically available genomes of human clinical strains. The AEEC cattle strains displayed synonymous SNP genotypes with *stx*_2_-positive sequence type 29 (ST29) human O26:H11 strains, while *stx*_1_ ST21 human and cattle strains clustered separately, demonstrating the close phylogenetic relatedness of these Shiga toxin-negative AEEC cattle strains and human clinical strains. With the exception of seven *stx*-negative strains, five of which contained *espK*, three *stx*-related SNPs differentiated the STEC strains from non-STEC strains, supporting the hypothesis that these AEEC cattle strains could serve as a potential reservoir for new or existing pathogenic human strains. Our results support the idea that targeted amplicon sequencing for SNP genotyping expedites strain identification and genetic characterization of E. coli O26:H11, which is important for food safety and public health.

## INTRODUCTION

Shiga toxin-producing Escherichia coli (STEC) O26 strains have been associated with large food-borne outbreaks worldwide and can result in severe human illness (1–3). Zoonotic pathogens, such as STEC O26, can contaminate produce, meat, and dairy products through the transmission from ruminants, such as cattle (4, 5). Enterohemorrhagic E. coli (EHEC) O26:H11, a subgroup within STEC that causes severe human disease, is believed to have originated from attaching and effacing E. coli (AEEC) O26:H11 through the acquisition of a Shiga toxin-encoding gene (6). AEEC strains cause typical attaching and effacing lesions in the intestinal mucosa and are characterized by the absence of a Shiga toxin gene (*stx*) and the presence of an intimin gene (*eae*), carried on the locus of enterocyte effacement (LEE) (6, 7).

A new highly pathogenic EHEC O26:H11 clone containing only *stx*_2a_ has previously been described, with an increased virulence and disease severity compared to O26:H11 strains containing *stx*_1_ alone or in combination with *stx*_2_ (8). Since its emergence in Germany in the early 1990s, this new *stx*_2a_ clone has disseminated around the world (1, 4, 8–18). Multilocus sequence typing has shown that this clone belongs to sequence type 29 (ST29) (8), which is associated with severe human disease, while the phylogenetically related group ST21 is associated with less-severe disease (6, 10, 19).

More recently, a new subpopulation of pathogenic O26:H11 strains harboring *stx*_2a_ or *stx*_2d_, belonging to ST29, has been identified in hemolytic-uremic syndrome (HUS) patients in France (19). These *stx*_2_-positive strains differ from the characteristic typical O26:H11 EHEC strains (20). Indeed, while they possess a *stx* gene and the *eae*-beta allele, they lack other genetic features, such as the effector translocated by the type III secretion system, *espK*, and contain a different allele of the aerobic respiratory control protein A (*arcA*) (19). They also exhibit a distinct clustered regularly interspaced short palindromic repeat (CRISPR) locus. To detect this French O26:H11 subpopulation, the CRISPR PCR marker SP_O26-E was developed (19). Surprisingly, AEEC O26:H11 strains of bovine fecal origin isolated in the United States have been observed to contain virulence-associated genes and the CRISPR marker SP_O26-E that is specific for the detection of the new French clone (21).

The evolutionary phylogenetic relationship of EHEC O26:H11/H^−^ has been investigated on strains of European origin employing whole-genome sequencing (WGS) in which four distinct clonal complexes (CCs) were observed using 48 phylogenetically informative single-nucleotide polymorphisms (SNPs) (22). The highly virulent German O26:H11 *stx*_2a_ clone was identified in a single CC, different from the hitherto described strains (22). To distinguish between strains containing a Shiga toxin-encoding gene and those strains lacking a Shiga toxin gene, the O-antigen gene cluster in E. coli O26:H11 has been investigated (23). Three SNPs were identified that, when used in combination, detected all *stx*-positive strains; one SNP captured a subset of strains containing only *stx*_2_ (23).

High-throughput next-generation sequencing (NGS) technologies have created a gateway into genomic research through accelerated genetic discoveries. WGS has been shown to be a powerful and informative tool for SNP discovery in which markers that provide genetic variations for hypothesis investigation are identified (22, 24–27). Although performing WGS on individual bacterial strains is becoming more cost-effective (28–30), sequencing many strains in the discovery process may be unnecessary (24), and computational genomics and bioinformatics pipelines can create a bottleneck in data analysis (24, 31). To circumvent these issues, targeted amplicon sequencing of SNPs using NGS can be used as a powerful approach for the rapid phylogenetic classification and characterization of several hundred to thousands of bacterial strains.

Extensive investigation into the phylogeny of STEC O26:H11 strains has been performed (22, 26, 32); however, there are missing links in how Shiga toxin-negative, *eae*-positive AEEC strains are related to pathogenic O26:H11 strains. Here we describe the investigation of the phylogenetic relationship and genetic diversity of AEEC O26:H11 bovine strains by the use of a high-throughput method for preparing targeted SNP amplicons for NGS.

## MATERIALS AND METHODS

### Bacterial strains.

E. coli O26:H11 bovine fecal strains (*n* = 178), isolated from May to July 2011 in cattle originating from a commercial feedlot in the United States housing 17,148 cattle in 40 pens (33), were investigated. These strains have previously been studied for their association with select virulence-associated and O26 serogroup-specific molecular markers along with the amplification of their CRISPR 1 and 2a loci. These strains displayed 37 unique genetic profiles, diversity types (DTs), determined by the combination of molecular markers and CRISPR alleles (Table 1) (21). Out of the strains investigated, 168 consisted of Shiga toxin-negative and *eae*-beta-positive strains. This combination of characteristics classifies these strains as AEEC (6, 7). Of these strains, 161 displayed the characteristic profile of the recently identified new French *stx*_2a_ and *stx*_2d_ clone containing the CRISPR PCR marker SP_O26-E (DTs 9 to 37) (19, 21). An additional five strains were Shiga toxin positive, *eae*-beta positive, and *espK* positive, classifying them as EHEC O26:H11 (DTs 2 and 3). Also, five strains were Shiga toxin negative, *eae*-beta positive, and *espK* positive (DT 4), classifying them as EHEC-like O26:H11. One strain was *stx*_1_ positive and *espK* negative and contained SP_O26-E (DT 1) (21).

**TABLE 1:** Single-nucleotide polymorphism clonal complexes, Shiga toxin-associated alleles, and molecular characteristics of cattle O26:H11 strains

DT^*a*^^,^^*b*^	No. of isolates	SNP CC^*c*^	STEC allele^*d*^	SNP in the following gene^*g*^:			Presence or absence of the following gene:					CRISPR1 allele^*a*^	CRISPR2a allele^*a*^^,^^*e*^	CT^*a*^^,^^*f*^
				*rmlA* STEC (G → T)	*wzx* STEC (T → G)	*fnl-1* STEC (G → A)	*stx*_1_^*a*^	*espK*^*a*^	*ehxA*^*a*^	*arcA2*^*a*^	SP_O26-E^*a*^			
1	1	1	0	G	T	G	+	−	−	−	+	29	67	3
2	3	3	1	**T**	T	**A**	+	+	+	+	−	11	160	1
3	2	4	1	**T**	T	**A**	+	+	+	+	−	9	159	2
4	5	1	1	G	**G**	G	−	+	+	+	−	29	4	4
5	1	1	0	G	T	G	−	−	−	−	−	U	24	18
6	1	1	0	G	T	G	−	−	−	−	−	29	162	6
7	3	1	0	G	T	G	−	−	−	−	−	131	72	14
8	1	1	0	G	T	G	−	−	−	−	−	29	67	3
9	7	1	0	G	T	G	−	−	−	−	+	29	67	3
10	1	1	0	G	T	G	−	−	−	−	+	116	U-4	22
11	8	1	0	G	T	G	−	−	−	−	+	116	70	13
12	2	1	1	G	**G**	G	−	−	−	−	+	29	67	3
12	62	1	0	G	T	G	−	−	−	−	+	29	67	3
13	3	1	0	G	T	G	−	−	−	−	+	130	67	17
14	1	1	0	G	T	G	−	−	−	−	+	29	U-5	23
15	1	1	0	G	T	G	−	−	−	−	+	29	U-6	24
16	2	1	0	G	T	G	−	−	−	−	+	29	67	3
17	2	1	0	G	T	G	−	−	−	−	+	29	67	3
18	1	1	0	G	T	G	−	−	−	−	+	29	67	3
19	14	1	0	G	T	G	−	−	−	−	+	11	70	5
20	13	1	0	G	T	G	−	−	−	−	+	125	161	7
21	8	1	0	G	T	G	−	−	−	−	+	126	161	8
22	1	1	0	G	T	G	−	−	−	−	+	116	70	13
23	1	1	0	G	T	G	−	−	−	−	+	129	72	15
24	1	1	0	G	T	G	−	−	−	−	+	129	70	16
25	1	1	0	G	T	G	−	−	−	−	+	11	70	5
26	9	1	0	G	T	G	−	−	−	−	+	125	161	7
27	2	1	0	G	T	G	−	−	−	−	+	126	161	8
28	1	1	0	G	T	G	−	−	+	−	+	126	161	8
29	1	1	0	G	T	G	−	−	−	−	+	126	161	8
30	3	1	0	G	T	G	−	−	−	−	+	120	67	9
31	1	1	0	G	T	G	−	−	−	−	+	120	159	10
32	3	1	0	G	T	G	−	−	−	−	+	120	24	11
33	1	1	0	G	T	G	−	−	−	−	+	124	160	12
34	7	1	0	G	T	G	−	−	−	−	+	120	U-1	19
35	1	1	0	G	T	G	−	−	−	−	+	120	U-2	20
36	1	1	0	G	T	G	−	−	−	−	+	124	U-3	21
37	1	1	0	G	T	G	−	−	+	−	+	120	U-2	20

a These E. coli O26:H11 strain characteristics originated from previously published results (21) which investigated the diversity and molecular characteristics of the strains in this study.

b The diversity type (DT) classification previously reported for the O26:H11 strains was determined through the combination of molecular markers and CRISPR alleles (21).

c SNP clonal complex (SNP CC) for which E. coli O26:H11 strains were classified based on the 48 phylogenetically informative SNPs previously identified in the SNP discovery study (22).

d Allele pattern of O-antigen genes for the presence of either the *stx*_1_ or *stx*_2_ STEC-associated allele.

e CRISPR alleles denoted with a U indicate that the CRISPR amplicon was unable to be amplified.

f Each unique combination of CRISPR1 and CRISPR2a alleles was assigned a CRISPR type (CT).

g Right arrows indicate the nucleotide SNP change; letters in bold indicate that the STEC allele is displayed.

### Targeted single-nucleotide polymorphisms.

Oligonucleotides were designed to target 51 previously identified SNPs (22, 23). Forty-eight SNPs, previously determined to be phylogenetically informative by Bletz et al. (22), were investigated in the bovine O26:H11 strains to determine the phylogenetic association of these strains to EHEC O26 strains of human origin that group into four distinct SNP clonal complexes (22). Three additional SNPs, located in the O-antigen gene cluster, which had been determined to differentiate Shiga toxin-positive strains from Shiga toxin-negative strains were investigated (23). A combination of STEC allele *rmlA* or *fnl-1* with *wzx* was observed to capture all *stx*-positive O26 strains with *wzx* capturing a subset of strains that contained only *stx*_2_ (23).

SNPs with reference SNP genotypes are provided in Table S1 in the supplemental material. E. coli O26:H11 strain 11368 NCBI reference sequence NC_013361.1 (GenBank accession no. AP010953.1) was used as the reference strain (34).

### Target-specific primer characteristics.

The Fluidigm Access Array system (Fluidigm, South San Francisco, CA) provides an expedited workflow from strain to targeted amplicons that are sequence ready and compatible on multiple NGS platforms. The Access Array two-primer target-specific PCR amplification generates 2,304 PCRs on a single integrated fluidic circuit (IFC) through the combination of 48 samples with 48 target-specific primer pairs (35). The *Access Array System for Illumina Sequencing Systems User Guide* (36) was used with the modifications described below to perform the two-primer target-specific PCR amplification on the 48.48 Access Array integrated fluidic circuit. Targeted next-generation sequencing with the Fluidigm Access Array amplifies a short genomic region of 100 to 150 bp in length with a final product of 150 to 200 bp. Oligonucleotides were designed to target the absolute genome positions of the previously identified 51 SNPs with the targeted SNP to be placed in the center of the final amplified product (see Table S2 in the supplemental material). Oligonucleotides were designed with a melting temperature of 59 to 62°C and optimum temperature of 60°C. Primers were selected with required specifications using Primer3Plus (37) and OligoCalc, an oligonucleotide properties calculator (38).

Common sequence 1 (CS1) (5′-ACACTGACGACATGGTTCTACA-3′) and common sequence 2 (CS2) (5′-TACGGTAGCAGAGACTTGGTCT-3′) universal primer sequences, required for Illumina MiSeq amplicon tagging and indexing, were added to the 5′ ends of all target-specific forward (TSF) and reverse (TSR) primers, respectively. The *Fluidigm Access Array - Generate Tagged Primers Workbook* (39) was used to add universal sequence tags to the target-specific primers (see Table S3 in the supplemental material). Primers were purchased from Eurofins Genomics (Ebersberg, Germany).

### Primer validation.

Target-specific primer validation for two-primer reactions on the 48.48 Access Array IFC was modified to include target-specific primers and was performed in a 96-well PeqSTAR thermocycler. Two E. coli O26:H11 strains and water were used as positive and negative controls for primer validation, respectively. The validation master mix solution with final concentrations contained 1× FastStart high-fidelity reaction buffer without MgCl_2_ (Roche, Meylan, France), 4.5 mM MgCl_2_ (Roche), 5% dimethyl sulfoxide (DMSO) (Roche), 200 μM each PCR-grade nucleotide mix (Roche), 1× Access Array loading reagent (Fluidigm, South San Francisco, CA), tagged TSF and TSR primers (200 nM each), 10 ng/μl DNA, 0.05 U/μl FastStart high-fidelity enzyme blend (Roche), molecular grade water to a final volume of 10 μl. The thermocycler program run with 35 cycles was performed as recommended in the Fluidigm targeted sequencing primer validation protocol (36) and provided in Table S4 in the supplemental material. PCR products were checked for each PCR using the FlashGel system (Lonza, France) with the FlashGel DNA 50-bp to 1.5-kb high-concentration marker to ensure that only one PCR product of the correct size was generated for each target.

Three duplex primer pairs were validated as previously described with substitution of 1 μl of molecular grade water with 0.5 μl of each additional primer. Primer pairs selected for duplex were first compared on the Thermo Fisher Scientific multiple primer analyzer (https://www.thermofisher.com/us/en/home/brands/thermo-scientific/molecular-biology/molecular-biology-learning-center/molecular-biology-resource-library/thermo-scientific-web-tools/multiple-primer-analyzer.html). Duplexing primer pairs allowed 51 targeted SNPs to be run on a single 48.48 Access Array IFC for a total of 2,448 amplicons generated on each 48.48 Access Array IFC. Primers combined in duplex for targeting SNPs were *fruR* with *murC*, *ybcF* with *sdhB*, and *ybjG* with *hyaE*.

### 48.48 Access Array IFC workflow.

**FIG 1:**
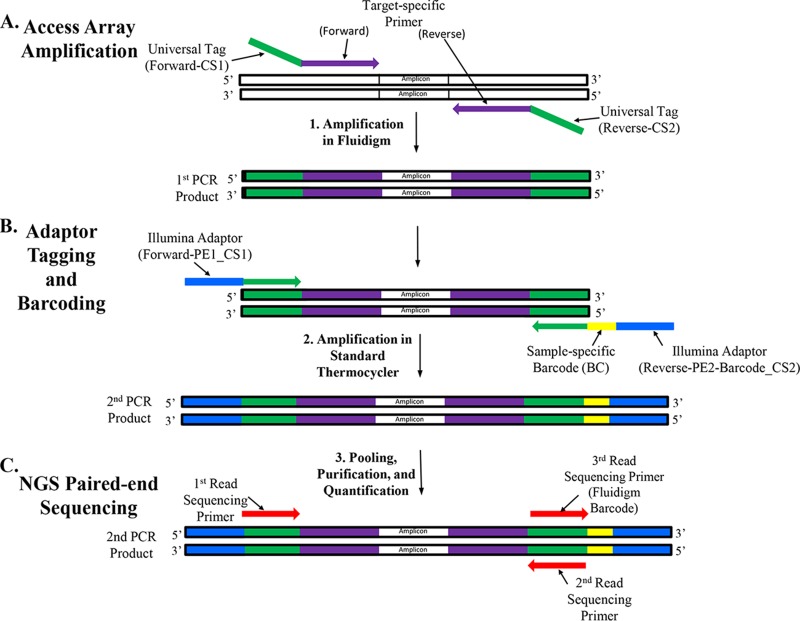
48.48 Access Array high-throughput workflow. (A) The first amplification on the Fluidigm system resulting in targeted amplicons containing universal tags on both ends. (B) A standard thermocycler is used to perform the second PCR, adding Illumina-specific adaptors and a sample-specific barcode. (C) Paired-end next-generation sequencing (NGS) is performed incorporating first-read, second-read, and third-read sequencing primers to read the sample-specific barcode.

The two-primer target-specific PCR amplification was performed at the iGenSeq Genotyping and Sequencing Core facility at the ICM Brain and Spine Institute (Paris, France) on the 48.48 Access Array IFC according to the manufacturer's recommendations (40). The Fluidigm 48.48 Access Array workflow for Illumina sequencing involves two independent PCRs as follows (Fig. 1). First, the 48.48 Access Array IFC is primed on a pre-PCR IFC controller AX (Fluidigm), 48 separate primer solutions are added to individual primer inlets, and 48 DNA sample mix solutions are added to individual sample inlets. The pre-PCR IFC controller AX combines each of the 48 primers and 48 samples into 2,304 separate microreaction chambers. The 48.48 Access Array IFC is then placed onto the FC1 cycler (Fluidigm) to complete the first thermocycler reaction (Fig. 1A). After thermal cycling, an Access Array harvest solution is added to the Access Array IFC and moved to a post-PCR IFC controller AX to perform the Harvest (151×) run script. In this step, all the amplicons generated for each sample are collected in the corresponding sample inlet. The tagged target-specific PCR amplicons are then transferred from the Access Array IFC to a 96-well PCR plate, resulting in sample-specific pooled amplicons. Four 48.48 Access Array IFCs were used in this study. For proper loading of the Access Array IFC, water was added in place of DNA in sample solution to combine with primer solution for incomplete sample plates.

In the second PCR, performed on a conventional thermocycler, Illumina sequence-specific adaptors with individual sample barcodes on the reverse primer were attached to the amplicons produced in the first PCR (39) (Fig. 1B). The Fluidigm Access Array barcode library for Illumina sequencers 384 (single-direction) kit (PN 100-4876; Fluidigm) barcode numbers 1 to 178 were used for amplicon tagging. The Illumina-specific primer sequences are as follows: forward (PE1_CS1), 5′-AATGATACGGCGACCACCGAGATCTACACTGACGACATGGTTCTACA-3′; and reverse (PE2_Barcode_CS2), 5′-CAAGCAGAAGACGGCATACGAGAT-(barcode)-TACGGTAGCAGAGACTTGGTCT-3′. Each DNA sample was assigned a unique barcode sequence, which is used for demultiplexing samples downstream.

The PCR products generated were qualified using a LabChip Gx reader (PerkinElmer Inc.) (see Fig. S1A and S1B in the supplemental material). All PCR products were then pooled to create a single product library. The quality of the pooled library was checked on a LabChip GX reader (PerkinElmer Inc.), and the library concentration was determined on the QuantiFluor system (Promega) (Fig. S1C).

### Amplicon sequencing using the Illumina MiSeq.

Custom Access Array primers (FL1 and FL2) are incorporated to read the amplicons generated from the Access Array IFC. FL1 and FL2 primer mixes were prepared according to the *Access Array System for Illumina Sequencing Systems User Guide* (36). The FL1 primer mix was prepared by combining CS1 oligonucleotide (5′-A+CA+CTG+ACGACATGGTTCTACA-3′) and CS2 oligonucleotide (5′-T+AC+GGT+AGCAGAGACTTGGTCT-3′). The FL2 primer mix combines the CS1rc oligonucleotide (5′-T+GT+AG+AACCATGTCGTCAGTGT-3′) and CS2rc oligonucleotide (5′-A+GAC+CA+AGTCTCTGCTACCGTA-3′); locked nucleic acid (LNA) nucleotides are preceded by a + sign; oligonucleotides were synthesized by TIB MOLBIOL (Berlin, Germany).

Denaturation and dilution of the Access Array pooled library was performed according to the Illumina preparing libraries for sequencing on the MiSeq protocol (41). The library was first diluted to 4 nM based on the QuantiFluor concentration and the average length (in base pairs) of the libraries (calculation provided in Table S5 in the supplemental material). The denatured DNA for 4 nM library procedure was performed and diluted to a final concentration of 8 pM.

The denatured and diluted sample library was sequenced on an Illumina MiSeq instrument as a two 150-bp paired-end runs (MiSeq flow cell, v2 reagents) according to the *Illumina MiSeq System User Guide* (42) (Fig. 1C) with the following modifications. Sequencing was performed with an index read length of 10 cycles to read all 10 bases of the Fluidigm indexes (barcodes). If only 96 of the Fluidigm barcodes are used, i.e., 96 samples, only the first 8 bases of the indexes would need to be read.

### Bioinformatics analysis.

Sequencing reads were demultiplexed according to the sample-specific barcodes on the Illumina MiSeq sequencer. A reference sequence was created by concatenating the sequences of the 51 amplicons from the reference *stx*_1_-positive strain 11368 NCBI reference sequence NC_013361.1 (GenBank accession no. AP010953.1), and sequence reads for each E. coli strain were mapped to this reference sequence using the CLC Genomics Workbench software version 7.5.1 (Qiagen, Aarhus, Denmark). Sequences were trimmed to ensure removal of Access Array barcodes with a quality score of 0.05 and a minimum read length of 15. The Basic Variant Detection tool in the CLC Genomics Workbench was used to identify and output SNP variants present. An R script (version 3.0.2) was written to reformat the CLC output of SNP variants and concatenate nucleotides at each SNP position for analysis. R script has been made publically available at https://github.com/sarahannison.

A minimum spanning tree was created by concatenating the 48 SNPs proposed by Bletz et al. (22) to identify the relationship of our Shiga toxin-negative cattle strains to four previously observed, clinically and phylogenetically associated, clonal complexes (CCs). Ten unique SNP profiles from 120 EHEC O26 isolates, which led to the designation of four CCs, were observed in the Bletz et al. SNP discovery study (22), and a representative strain was designated for each observed SNP genotype. The SNP genotypes of these 10 strains, 1226/65, 1557/77 (DEC10c), 2245/98 (HUSEC013), 5080/97 (HUSEC014), 126814/98 (HUSEC015), 5028/97 (HUSEC016), 3319/99 (HUSEC017), 1530/99 (HUSEC018), 1588/98 (HUSEC019), and 3271/00 (HUSEC020) (22), were used to guide the construction of the tree and determine the relationship of the cattle O26:H11 strains in this study to the four previously identified CCs. The minimum spanning tree was created in Splitstree (43) (version 4.13.1) with character inputs, Jukes Cantor, and MinSpanning Network for Distances.

### SNP analysis of O26:H11 human clinical strains.

Publically available whole-genome sequences of seven human clinical O26:H11 strains, originating from the United States, were investigated for the 48 phylogenetically informative SNPs and the three O-antigen STEC allele SNPs. Strains included 2009C-3612 (NCBI accession no. JHGZ00000000), 2009C-3689 (GenBank accession no. JHGX00000000), 2009C-3996 (GenBank accession no. JHGV00000000), 2009C-4747 (GenBank accession no. JHGM00000000), 2009C-4760 (GenBank accession no. JHGK00000000), 2009C-4826 (GenBank accession no. JHGI00000000), (44), and CFSAN001629 (GenBank accession no. AMXO00000000) (45). The Shiga toxin gene present (https://www.patricbrc.org/) (46) and multilocus sequence type (MLST) of each strain was determined (http://mlst.warwick.ac.uk/mlst/dbs/Ecoli) (47). The reference nucleotide sequence containing each SNP was aligned with the publically available whole-genome sequence for each strain by using a local BLAST (version 2.2.29) (48) script (BLoPv2) that was developed in-house. This script utilizes a single text file containing all of the targeted nucleotide sequences in individual Fasta format and a local BLAST script to identify the degree of similarity with each sequence provided into a single .html output file. As a result, the nucleotide at each SNP position was quickly identified.

## RESULTS

### SNP genotyping by next-generation sequencing.

Of the 178 E. coli O26:H11 bovine strains that were investigated for the 48 phylogenetically informative SNPs and 3 Shiga toxin-associated SNPs, one strain, strain 4850A (see Fig. S1B in the supplemental material), did not generate the targeted library size and was excluded from the analysis. Qualification of the strain library is shown in Fig. S1C. The Illumina MiSeq platform sequencer produced 150-bp paired-end reads yielding 3.0 × 10^6^ total reads with approximately 16,949 reads per sample and 330 reads per targeted locus. Of the 9,027-targeted loci, 61 loci failed to be amplified or only one read for specific loci was produced (Table S6). Of the failed loci, 22 and 21 were observed in the *murC* and *rcsA* loci, respectively. Only two O26:H11 samples had two nondetermined loci, and no more than one locus was undetermined in all other samples.

### Classification of AEEC O26:H11 bovine strains according to phylogenetically informative SNPs.

**FIG 2:**
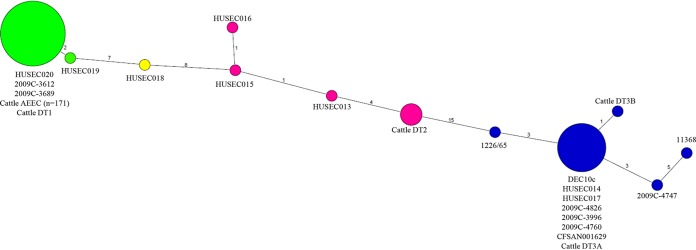
Minimum spanning tree of 48 SNPs used to identify four clonal complexes and relationship of cattle Shiga toxin-negative O26:H11 strains. Clonal complex SNPs were previously proposed by Bletz et al. (22). Five Shiga toxin-positive cattle O26:H11 strains are included for reference (21). The four SNP clonal clusters (SNP CCs) are represented by different colors as follows: SNP CC1 is green, SNP CC2 is yellow, SNP CC3 is pink, and SNP CC4 is blue. The small numbers on the lines connecting the circles represent the differing number of SNPs between two nodes. The number of isolates present within a node are reflected by the size of the circle, with the exception that the graphical representation of the AEEC cattle O26:H11 node is presented at 5% of the actual size. In the Bletz minimum spanning tree (shown in Fig. 2 of reference 22), HUSEC015 and HUSECO16 are displayed in the same node; however, they differ in one SNP, creating a new node. This figure was created with Splitstree (version 4.13.1) (43) characters > Jukes Cantor distances > MinSpanning Network for Distances.

Concatenated SNP genotypes of the 48 phylogenetically informative SNPs were created for all cattle O26:H11 strains, and a minimum spanning tree that included the reference strains (22) for each clonal complex was then constructed (Fig. 2). SNP clonal complex 1 (CC-1) contained strains that were either *stx*_1_ ST29 or *stx*_2_ positive and belonged to ST29 or a single-locus variant of ST29, ST396. It has previously been identified that a large majority of these AEEC O26:H11 cattle strains also belong to ST29 (21). Notably, a few *stx*_2a_ ST29 strains that resulted in HUS also clustered in CC-1 in the original SNP discovery study (22). Analysis of the 48 phylogenetic informative SNPs determined that all of the AEEC O26:H11 strains (*n* = 171) displayed the *stx*_2a_ ST396 HUSEC020 strain SNP genotype (see Table S7 in the supplemental material) and clustered into CC-1, shown in green in Fig. 2. Thus, the bovine AEEC O26:H11 strains studied here appear related to this cluster of strains. The *stx*-positive, SP_O26-E-positive strain, displaying the molecular marker and CRISPR allele combination for diversity type 1 (DT 1) (21) (Table 1) also displayed this CC-1 SNP genotype.

Three *stx*-positive strains, belonging to DT 2, clustered within CC-3 (shown in pink in Fig. 2), exhibited a four-SNP difference from strain HUSEC013, and differed in 15 SNPs from CC-4 strain 1226/65. One DT 3, *stx*-positive strain displayed the CC-4 (shown in blue in Fig. 2) SNP genotype synonymous with strains DEC10c, HUSEC014, and HUSEC017. The other DT 3 strain, also grouped in CC-4, differed from this SNP profile at one SNP position.

### Nucleotide polymorphisms discriminating Shiga toxin-positive O26:H11 strains from Shiga toxin-negative O26:H11 strains.

The three O-antigen SNPs investigated differentiated the STEC strains from non-STEC strains, with the exception of seven *stx*-negative strains that displayed the *wzx stx*_2_ STEC allele (Table 1). Interestingly, five of these strains also contain *espK*, a virulence-associated effector translocated by the type III secretion system, which substantiates their classification as EHEC-like strains (49). One *stx*-positive strain, belonging to DT 1, was also not identified using the STEC-associated polymorphisms. However, this strain was negative for *espK* and all other virulence-associated markers that have previously been investigated (21).

### SNP genotyping of human clinical O26:H11 strains.

Two human strains, 2009C-3612 and 2009C-3689, were identified to harbor only *stx*_2a_ and belonged to MLST ST29. These strains exhibited synonymous SNP genotypes with the HUSEC020 strain (see Table S7 in the supplemental material), belonging to CC-1 (Fig. 2). The O-antigen *stx*_2_ STEC-associated allele *wzx* was also observed in these strains. The additional five strains were identified as *stx*_1_ and belonging to the MLST ST21. These human clinical strains clustered into CC-4. Four strains, 2009C-4826, 2009C-3996, 2009C-4760, and CFSAN001629, displayed the same SNP genotype profile as that of strains HUSEC017, HUSEC014, and DEC10c. Strain 2009C-4747 differed at three SNP positions from this group and at an additional five SNPs from the O26:H11 reference strain 11368 (Table S7).

## DISCUSSION

### Investigation into genetic characteristics further substantiates similarities between human and bovine O26:H11 strains.

In this study, 51 primer pairs were designed to target 51 previously identified SNPs for next-generation sequencing via a high-throughput targeted amplicon sequencing approach for phylogenic inference of AEEC O26:H11. The 48 phylogenetically informative SNPs investigated in this study had been observed to form four distinct SNP CCs in EHEC O26 strains and were used to infer an evolutionary model for EHEC O26 (22).

CRISPRs are composed of partially palindromic DNA repeats separated by unique spacers created from exogenous DNA, either from viruses or plasmids, entering the bacterial cell (50, 51). Due to recognition mechanisms of the CRISPR complex, future invasion of this exogenous DNA into the bacterial chromosome is inhibited, therefore creating an adaptive immunity (52). It has also been observed that CRISPR loci are conserved among phylogenetically related bacterial strains (53–55). Previous investigation into the CRISPR 1 and 2a loci of these ST29 AEEC cattle isolates identified that a majority of strains displayed the CRISPR 1 allele 29 and CRISPR 2a allele 67 (21); this CRISPR allele combination had also been previously observed in a *stx*_2_-positive O26 cattle strain (54). The same allele combination has also been identified in the pathogenic human French ST29 *stx*_2_ clone displaying the CRISPR PCR marker SP_O26-E (19). More recently, the same CRISPR allele combination was identified in O26 strains isolated from beef products in processing plants (55), providing evidence that beef products may serve as a route of transmission to humans. The CRISPR 1 and 2a alleles for the U.S. human strains, 2009C-3612 and 2009C-3689, were determined using a CRISPR allele database (21). Strain 2009C-3612 displayed CRISPR 1 allele 29 and CRISPR 2a allele 4, while strain 2009C-3689 displayed CRISPR 1 allele 29 and a CRISPR 2a allele that was not observed in the published database (21). However, this allele, containing the spacer combination 7-11-12-13-14-15, is related to allele 4, spacer combination 7-8-9-10-11-12-13-14-15, given that they share a pool of spacers. The five DT 4 AEEC cattle strains in this study contain the CRISPR type (CT) representing a combination of the CRISPR 1 and 2a alleles, synonymous with that of the human 2009C-3612 strain, CT 4. These cattle strains were also negative for the SP_O26-E CRISPR PCR marker; this marker was also absent from *stx*_2a_ French O26 ST21 and ST29 strains that contained the CRISPR 2a allele 4 (19). Interestingly, the CRISPR 2a allele 4 was previously observed as the most common allele identified in a collection of O26:H11 *stx*_1_ strains (54). Investigation of the CRISPR locus identifies similarities and shared characteristics among cattle strains and human clinical strains.

### Expanding the classification of O26:H11 SNP clonal complexes.

Early investigation into the new German EHEC O26 clone (8) identified EHEC O26 ST29 strains carrying only *stx*_2a_ to display the plasmid gene profile *ehxA* positive (EHEC hemolysin), *katP* negative (catalase-peroxidase), *espP* negative (serine protease EspP), and *etpD* positive (type II secretion system effector), while the absence of *ehxA* and *etpD* was observed on the plasmid of the new French *stx*_2a_ subpopulation (19). With the exception of seven strains (3.95%), all AEEC O26:H11 strains investigated in this study lacked *ehxA*, and 161 strains (90.96%) displayed the characteristic profile of the French clone (21). The clustering of the AEEC strains investigated here in combination with the molecular similarities observed with *stx*_2a_ ST29 strains demonstrates that the SNP CCs 1 and 2 may not accurately identify subsets of *stx*_2a_ ST29 strains. However, the SNP genotypes and virulence-associated markers present within these cattle AEEC O26:H11 strains and shared with pathogenic human strains, both of U.S. and European origin, provide evidence of the relatedness of these strains. The respective inclusion of three and two cattle O26:H11 strains into CC-3 and CC-4, along with the U.S. human clinical isolates into CC-4 was expected, given that these strains are *stx*_1_ positive only and belong to ST21.

### Overcoming shortfalls in SNP discovery studies.

We acknowledge that the SNPs used in this genotyping assay, with the exception of one strain, were identified in strains of European origin, while the cattle strains investigated here belong to a U.S. sample population, and the inclusive and exclusive panel of strains for WGS and SNP discovery is important for identifying markers that are accurate for interpretation of phylogenetic information (24). However, inclusion of the U.S. human clinical isolates, of different Shiga toxin and MLST types, in this study substantiates our observations of the AEEC cattle O26:H11 strains being more closely related to highly pathogenic *stx*_2_ human strains than to *stx*_1_ strains.

A deeper phylogenetic relationship among and between strains could be achieved with the SNP genotyping assay through addressing limitations in the SNP discovery study. In the initial study, the 48 synapomorphic SNPs were chosen manually from four quarters of the E. coli O26:H11 strain 11368 chromosome (22). Some of these SNPs do not provide additional discriminatory information when used in combination with other specific SNPs in this assay and could be substituted for more phylogenetically distinct and informative SNPs. The locus tag combinations containing these less informative SNPs are provided in Table S8 in the supplemental material. Within these specific locus tag combinations, there is not an observed difference that would result in the discrimination of strains by the inclusion or exclusion of one locus tag with another. However, it is acknowledged that an addition of epidemiologically unrelated strains in this study could result in the necessary inclusion of the SNP positions above. This is supported by the findings presented here for the cattle DT3B strain at the *ycjF* SNP (ECO26_1890) position in which the observed nucleotide change at this position resulted in a SNP genotype specific to this strain, therefore resulting in a new node to be formed in the phylogenetic minimum spanning tree. Alternatively, rather than excluding these SNPs, the number of primer pairs can be multiplexed in the Access Array assay to increase those investigated. The Access Array system allows up to 10 primer pairs to be multiplexed together in a single primer inlet well. Indeed, the multiplexing capabilities of the Access Array assay can allow the investigation of up to 480 loci per sample, performing 23,040 PCRs on a single IFC.

### Evaluating the relatedness of AEEC O26:H11 cattle and human clinical O26:H11 strains.

As expected, the *stx*_2_-positive U.S. human clinical strains 2009C-3612 and 2009C-3689 exhibit only the *wzx* STEC-associated allele. The seven *stx*-negative O26:H11 cattle strains that displayed the *wzx* STEC-associated allele are in accordance with previous findings in which two strains carrying *espK* were captured (23) and with previous research identifying this gene as a relevant marker for identifying typical EHEC and EHEC-like strains (49). Further, these *stx*-negative EHEC-like strains may have contained or have the potential to acquire a Shiga toxin gene (6, 56, 57). The combination of *espK* and the *wzx* STEC-associated allele further substantiates that these *stx*-negative strains are phylogenetically related to the *stx*-positive strains. The other two *stx*-negative strains that displayed the *wzx* STEC allele belong to DT 12 and contain the SP_026-E CRISPR PCR marker. While the other two STEC-associated alleles, *rmlA* and *fnl-1*, were positively identified in all other human clinical and *stx*-positive cattle strains, it is important to recognize that the genes containing these discriminative polymorphisms, in addition to O26, are found in other serogroups (23).

### Application of targeted amplicon sequencing for SNP genotyping.

Targeted amplicon sequencing (TAS) provides a pipeline for data interpretation and analysis with limited bioinformatics background when used in combination with a commercially available variant detection program. In combination with NGS technology, TAS increases the throughput of samples, decreases cost, and expedites analysis compared to traditional sequencing methods (58, 59), thus allowing expedited data interpretation and an increased number of strains to be investigated. The application of TAS can be extended beyond the SNPs investigated in this study to include SNPs targeting the emergence of new clones, genomic regions related to physiology, outbreak investigations, and as application in studying antibiotic resistance. We acknowledge that this method is not a replacement for exploratory research aimed at the identification of unique characteristics but serves as a method to increase throughput and identification leading to phylogenetic classification or relatedness to human clinical isolates.

The use of the Access Array in the TAS procedure presented here provides an avenue of immense sample throughput due to its reduced cost benefits accomplished from the small amount of input DNA required, yielding small reagent volumes per sample, massive multiplexing capabilities, and reduced hands-on time. Furthermore, the primers designed to target the published SNPs investigated in this work can still be used for NGS of these SNPs using other library preparation methods, although they may be more labor-intensive. Alternatively, the primers can also be employed in a high-resolution melting (HRM) assay. This assay utilizes a HRM dye that binds double-stranded DNA (dsDNA) heteroduplexes to detect polymorphisms and generates melting curves of post-PCR products. The presence of different nucleotides affects the melting behavior, leading to the recognition of discernible differences in genotypes. Further, HRM laboratory equipment may currently be more widely available and multipurpose than that previously proposed by the TAS. To corroborate this application, we performed an HRM assay targeting a few SNPs on a subset of strains. A discernible difference was observed in the melting curves produced, indicating a nucleotide change within the targeted region (data not published). Further, the application of HRM has previously been applied in numerous scientific applications (60–64).

### Incorporating an Illumina adaptor-ligated library to increase sequence diversity and read quality.

Less than 1% of the targeted loci failed to be amplified in this study representing no more than one missing locus per strain, except for two strains. However, this missing SNP information had no negative consequences in strain classification for determining phylogenetic relationships of strains within and between clonal complexes. The occurrence of failed locus reads may have been due to the absence of said loci, such as *murC* and *rcsA*, in the genome of the E. coli samples. A technical problem due to the lack of sequence diversity could also have contributed to the absence of these results during sequencing, given that conserved sequences that contained very little difference in single nucleotide positions were investigated. To account for this, the Illumina PhiX control, an adaptor-ligated library of the small well-characterized PhiX virus genome, could be incorporated to balance the overall lack of sequence diversity and to increase the quality of sequence reads produced. This can be performed by diluting the denatured 20 pM PhiX library with prechilled HT1 to a final concentration of 12.5 pM. The PhiX control is then combined at 15% (90 μl) with the sample library (510 μl). Because the PhiX library is read with the Illumina sequencing primers, the custom primers, FL1 and FL2, are prepared as previously described and incorporated with the Illumina primers in the MiSeq cartridge to read the PhiX control during sequencing. It is important to note that these custom primers are not used in the MiSeq custom ports and the Sequencing Workflow Using Fluidigm FL1 and FL2 Sequencing Primers protocol in the *Access Array System for Illumina Sequencing Systems User Guide* (36) should not be followed if using the PhiX control in a sequencing run. Instead, the FL1 and FL2 reagents should be prepared for sequencing on the MiSeq instrument with PhiX according to the protocol described in Table S9 in the supplemental material.

### Conclusion.

Based on the genotypes observed within the 48 phylogenetically informative SNPs, these strains were classified into four previously identified clonal complexes (22). Of these strains, the U.S. cattle AEEC O26:H11 strains displayed SNP genotypes identical to those of *stx*_2_ ST29 U.S. human O26:H11 strains. The *stx*_1_ ST21 human and cattle strains clustered separately. Although the extent of the clinical outcome of the U.S. human strains is unknown, the similarities of these strains with the Shiga toxin-negative AEEC cattle strains demonstrate their close phylogenetic relatedness. The potential ability of Shiga toxin-negative E. coli strains to acquire a Shiga toxin (57, 65), and the presence of the STEC alleles in a few *stx*-negative cattle strains, further supports the relatedness of AEEC cattle strains to highly pathogenic human strains. NGS has provided an in-depth and robust method for investigation into the bacterial genome. Furthermore, analysis and classification of highly pathogenic bacteria using SNP assays can expedite the time to identification, which is important for food safety and human public health.

## Supplementary Material

Supplemental material
