# Supplementary material for: Targeted Amplicon Sequencing for Single-Nucleotide-Polymorphism Genotyping of Attaching and Effacing Escherichia coli O26:H11 Cattle Strains via a High-Throughput Library Preparation Technique
Source: Appl Environ Microbiol. 2016 Jan 7;82(2):640–9. doi: 10.1128/AEM.03182-15 (PMC4711113; doi:10.1128/AEM.03182-15)
Supplement: Supplemental material [file supp_82_2_640__index.html]

Targeted Amplicon Sequencing for Single-Nucleotide-Polymorphism Genotyping of Attaching and Effacing Escherichia coli O26:H11 Cattle Strains via a High-Throughput Library Preparation Technique — Supplemental material 

# Targeted Amplicon Sequencing for Single-Nucleotide-Polymorphism Genotyping of Attaching and Effacing Escherichia coli O26:H11 Cattle Strains via a High-Throughput Library Preparation Technique

## Supplemental material

- Supplemental file 1 -

  Individual strain and pooled quantification of strain library of PCR products generated (Fig. S1) and workflow protocol to prepare Illumina Miseq libraries spiked with the PhiX control (Table S9).

  PDF, 321K
- Supplemental file 2 -

  SNP genotypes and SNP changes (Table S1).

  XLSX, 15K
- Supplemental file 3 -

  Forward and reverse oligonucleotides to target the absolute genome position of 51 previously identified SNPs (Table S2).

  XLSX, 17K
- Supplemental file 4 -

  Access Array universal sequence tags for forward and reverse target-specific primers (Table S3).

  XLSX, 18K
- Supplemental file 5 -

  Fluidigm targeted sequencing primer validation thermocycler program (Table S4).

  XLSX, 9.2K
- Supplemental file 6 -

  Access Array pooled library dilution calculator for preparing libraries for Illumina sequencing (Table S5).

  XLSX, 11K
- Supplemental file 7 -

  Cattle *E.coli* O26:H11 SNP sequencing results (Table S6).

  XLSX, 48K
- Supplemental file 8 -

  *E. coli* O26:H11 cattle and U.S. human strain single-nucleotide-polymorphism genotypes (Table S7).

  XLSX, 13K
- Supplemental file 9 -

  Locus tag combinations of SNPs that produce homogeneous genotypes (Table S8).

  XLSX, 13K
