# Supplementary material for: Targeted Amplicon Sequencing for Single-Nucleotide-Polymorphism Genotyping of Attaching and Effacing Escherichia coli O26:H11 Cattle Strains via a High-Throughput Library Preparation Technique
Source: Appl Environ Microbiol. 2016 Jan 7;82(2):640–9. doi: 10.1128/AEM.03182-15 (PMC4711113; doi:10.1128/AEM.03182-15)
Supplement: Supplemental material [file AEM.03182-15_zam999116857so1.pdf]

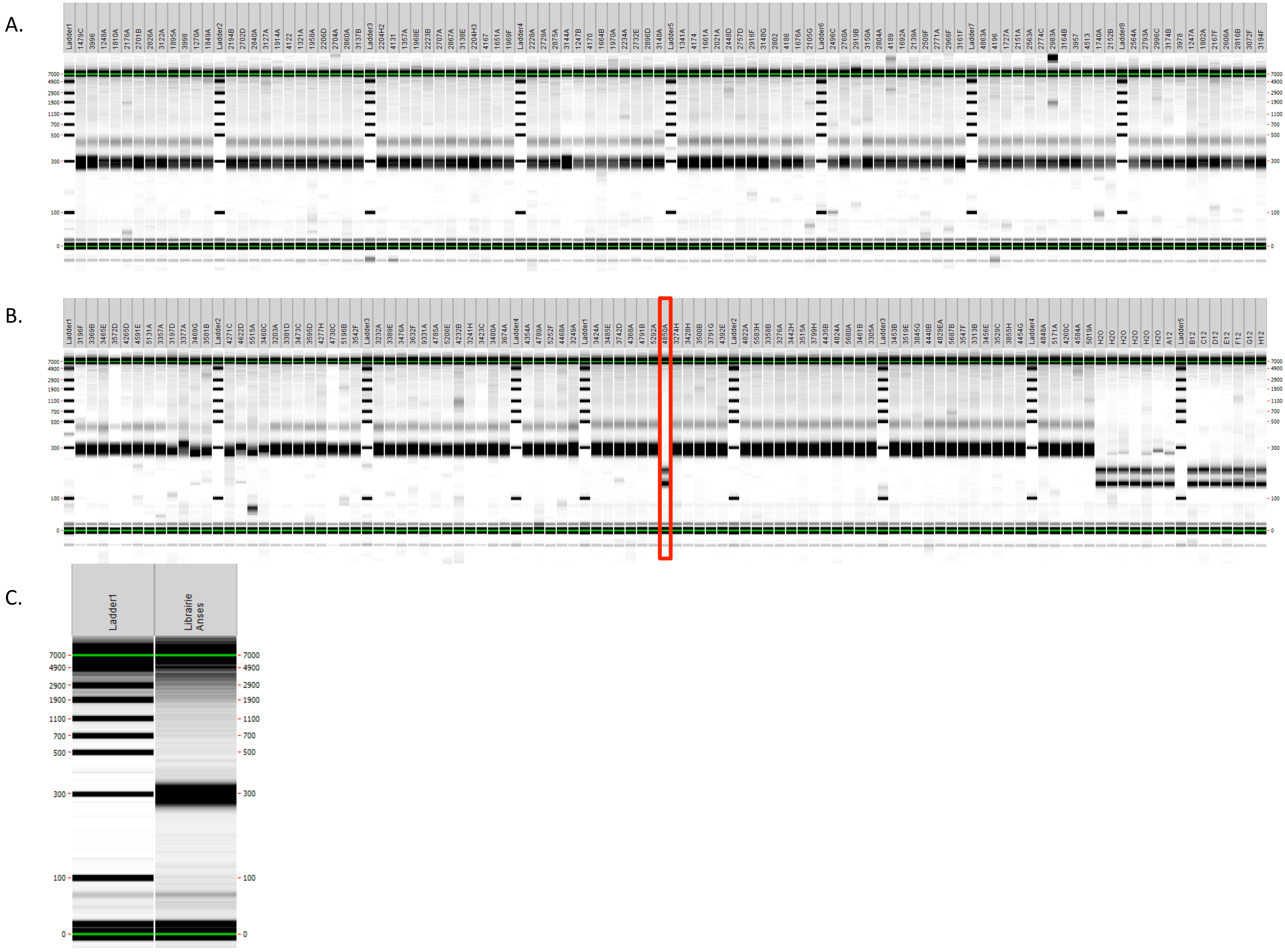
A.
Ladder1
1479C
3996
1248A
1810A
2176A
2701B
2826A
3122A
1895A
3998
1270A
1849A
Ladder2
2194B
2702D
2840A
3127A
1914A
4122
1321A
1958A
2206D
2704A
2860A
3137B
Ladder3
2204H2
4131
1357A
1968E
2223B
2707A
2867A
3138E
2204H3
4167
1651A
1969F
Ladder4
2228A
2729A
2875A
3144A
1247B
4170
1664B
1970A
2234A
2732E
2896D
3148A
Ladder5
1341A
4174
1661A
2021A
2448D
2757D
2916F
3148G
2802
4186
1676A
2105G
Ladder6
2496C
2760A
2919B
3150A
2804A
4189
1692A
2139A
2509F
2771A
2966F
3161F
Ladder7
4863A
4196
1727A
2151A
2563A
2774C
2983A
3164B
3957
4513
1740A
2152B
Ladder8
2564A
2793A
2996C
3174B
3978
1247A
1802A
2167F
2606A
2816B
3072F
3194F
7000
4900
2900
1900
1100
700
500
300
100
0
B.
Ladder1
3196F
3369B
3465E
3572D
4265D
4591E
5131A
3357A
3197D
3377A
3469G
3581B
Ladder2
4271C
4622D
5515A
3460C
3203A
3381D
3473C
3595D
4277H
4730C
5196B
3542F
Ladder3
3232A
3389E
3476A
3632F
9331A
4785A
5206E
4232B
3241H
3423C
3480A
3674A
Ladder4
4354A
4789A
5252F
4468A
3249A
Ladder1
3424A
3485E
3742D
4368A
4791B
5292A
4850A
3274H
3428H
3500B
3791G
4392E
Ladder2
4822A
5583H
3358B
3276A
3442H
3515A
3799H
4435B
4824A
5680A
3461B
3305A
Ladder3
3453B
3519E
3845G
4440B
4829EA
5687B
3547F
3313B
3456E
3529C
3855H
4454G
Ladder4
4848A
5171A
4260C
4584A
5019A
H2O
H2O
H2O
H2O
H2O
H2O
A12
Ladder5
B12
C12
D12
E12
F12
G12
H12
7000
4900
2900
1900
1100
700
500
300
100
0
C.
Ladder1
Librairie Anses
7000
4900
2900
1900
1100
700
500
300
100
0

Fig. S1

Fig S1.
Individual strain and pooled quantification of strain library of PCR products generated. Qualification of targeted amplicon products was generated using a Labchip Gx (Perkin-Elmer Inc.)

Table S9. Workflow protocol to prepare Illumina Miseq libraries spiked with the PhiX control.

This protocol describes a procedure and workflow on Illumina Miseq instrument for libraries spiked with PhiX control to read the read 1sequencing primer, the indexing primer, and the read 2 sequencing primer (for paired-end sequencing).

Reagent port	ILMN reagent	ILMN reagent description	FLDM primer
12	HP10	Read 1 primer mix	FL1 read 1/2 primer mix
13	HP12	Index primer mix	FL2 index primer mix
14	HP11	Read 2 primer mix	FL1 read 1/2 primer mix

Note: FL1 corresponds to CS1 and CS2 primers at 50 µM each and FL2 corresponds to CS1rc and CS2rc primer at 50 µM each.

1. Check volumes of the reagents contained in ports 12, 13 and 14
   a. Note: Illumina reagents may vary in volume and it is necessary to check the volume using a 1 ml Pasteur pipette
   b. Pipette out the reagents contained in ports 12, 13 and 14 into labeled tubes
   c. Transfer 693 µl of each of the 3 reagents into fresh labeled tubes and discard the remaining volume
   d. If there is not 693 µl volume from each reagent, add HT1 buffer to reach the specified volume
2. Add the FL1 primers to reagents from ports 12 and 14
   a. Retrieve the 50 µM FL1 primers.
   b. Add 7 µl of FL1 Primers at 50 µM per primer (CS1 and CS2) to the tubes containing 693 µl of reagents for both ports 12 and 14. The final concentration of the Fluidigm custom primers in this solution will be 0.5 µM.
   c. Vortex for 5 seconds and spin down.
   d. Add 700 µl of the reagents into each one of the ports 12 and 14.
3. Add the FL2 primers to reagents from port 13
   a. Retrieve the 50 µM FL2 primers.
   b. Add 7 µl of FL2 Primers at 50 µM per primer (CS1 and CS2) to the tube containing 693 µl of reagent for port 13. The final concentration of the Fluidigm custom primers in this solution will be 0.5 µM.
   c. Vortex for 5 seconds and spin down.
   d. Add 700 µl of the reagents into port 13.
